# Calculation and Analysis of Microstate Related to Variation in Executed and Imagined Movement of Force of Hand Clenching

**DOI:** 10.1155/2018/9270685

**Published:** 2018-08-27

**Authors:** Yunfa Fu, Jian Chen, Xin Xiong

**Affiliations:** School of Information Engineering and Automation, Kunming University of Science and Technology, Kunming 650500, China

## Abstract

**Objective:**

In order to investigate electroencephalogram (EEG) instantaneous activity states related to executed and imagined movement of force of hand clenching (grip force: 4 kg, 10 kg, and 16 kg), we utilized a microstate analysis in which the spatial topographic map of EEG behaves in a certain number of discrete and stable global brain states.

**Approach:**

Twenty subjects participated in EEG collection; the global field power of EEG and its local maximum were calculated and then clustered using cross validation and statistics; the 4 parameters of each microstate (duration, occurrence, time coverage, and amplitude) were calculated from the clustering results and statistically analyzed by analysis of variance (ANOVA); finally, the relationship between the microstate and frequency band was analyzed.

**Main Results:**

The experimental results showed that all microstates related to executed and imagined grip force tasks were clustered into 3 microstate classes (A, B, and C); these microstates generally transitioned from A to B and then from B to C. With the increase of the target value of executed and imagined grip force, the duration and time coverage of microstate B gradually decreased, while these parameters of microstate C gradually increased. The occurrence times of microstate B and C related to executed grip force were significantly more than those related to imagined grip force; furthermore, the amplitudes of these 3 microstates related to executed grip force were significantly greater than those related to imagined grip force. The correlation coefficients between the microstates and the frequency bands indicated that the microstates were correlated to mu rhythm and beta frequency bands, which are consistent with event-related desynchronization/synchronization (ERD/ERS) phenomena of sensorimotor rhythm.

**Significance:**

It is expected that this microstate analysis may be used as a new method for observing EEG instantaneous activity patterns related to variation in executed and imagined grip force and also for extracting EEG features related to these tasks. This study may lay a foundation for the application of executed and imagined grip force training for rehabilitation of hand movement disorders in patients with stroke in the future.

## 1. Introduction

The functional states of the brain constantly change even without external stimuli and tasks. EEG is a powerful tool to study the brain functional states because it has a high temporal resolution (millisecond level) and thus can detect instantaneous states with millisecond time resolution.

One of the methods to analyze the transient state of the brain is microstate analysis. Lehmann first proposed the concept of microstates in 1987 and decomposed the mu rhythm (8∼12 Hz) of multichannels resting state EEG signals into a finite number of discrete quasi-steady states—“microstates” [1]. Lehmann et al. believed that the state of the brain did not change continuously, and it was only in a very short period of time that brain activity may be thought to be stable; it then quickly jumped to the next stable state. EEG activity related to this brain activity was a quasi-state. They further assumed that the topographies of the brain's instantaneous potentials could reflect the instantaneous state of brain function and ultimately determined that the duration of this transient state was 80∼100 ms. Because the time scale of the microstate was similar to the speed of thought transformation, Lehmann et al. thought of the microstate as the “atom of thinking.”

Some studies have been conducted on microstate analysis in the resting state [2], such as the ones regarding behavioral modes [3, 4], personality types [5], mental disorders [6, 7], sleep stages [8, 9], and conscious perception [10]. Four typical EEG microstates, which correspond to the auditory network, visual network, salient network, and frontal-parietal network, were defined by microstate analysis in the resting state [11]. In addition, other studies have also been conducted on microstate analysis in task states, such as those regarding motor function, auditory stimulation [12, 13], visual stimulation [14], and driver's brain load during driving tasks [15, 16].

However, to our knowledge, EEG microstate analysis on executed and imagined grip force is yet to be carried out. Therefore, in this study, we explored how EEG microstates develop in 3 levels (high, medium, and low) of executed and imagined grip force modes.

## 2. Materials and Methods

### 2.1. Subjects and Training for Executed and Imagined Grip Force

In this study, 20 healthy subjects (12 males and 8 females, with an average age of 22.8 ± 5.1, undergraduate or graduate students) had EEG data collected under different grip force modes. All subjects were right-handed and had no history of sensorimotor disorder or mental disease affecting brain function; subjects gave signed informed consent and filled out a questionnaire of motor imagined ability before the experiment.

The subjects were asked to familiarize themselves with the experimental environment before the experiment and understand the requirements and principles of this experiment, thus improving subject compliance. In particular, it was necessary for the subjects to totally understand the mental activity paradigm of motor imagery and to select the suitable strategies for their motor imagery. In the training stage, subjects firstly used grip devices to perform the executed right-hand grip with 3 different forces: low grip force (4 kg), medium grip force (10 kg), and high grip force (16 kg). Here, subjects experienced the executed grip force. Then, subjects imagined the grip movement in the same way using kinesthetic imagination. Kinesthetic imagination asks subjects to imagine themselves performing some movement without any executed motion output, while visual imagination involves imagining watching others perform a certain exercise [17, 18]. In the study, the subjects were asked to feel or recall a movement in his/her brain at the first personal perspective without actual movement and to perform an amount of training for imagined movement of force of hand clenching until they were able to execute the motor imagery vividly in a controllable manner. After the training of the motor imagery, the vividness and controllability of motor imagery (movement imagery abilities) of force of hand clenching were measured by the Movement Imagery Questionnaire [19–23].

### 2.2. Experimental Setup, Paradigm, and Procedure

#### 2.2.1. Experimental Setup

**Figure 1:**
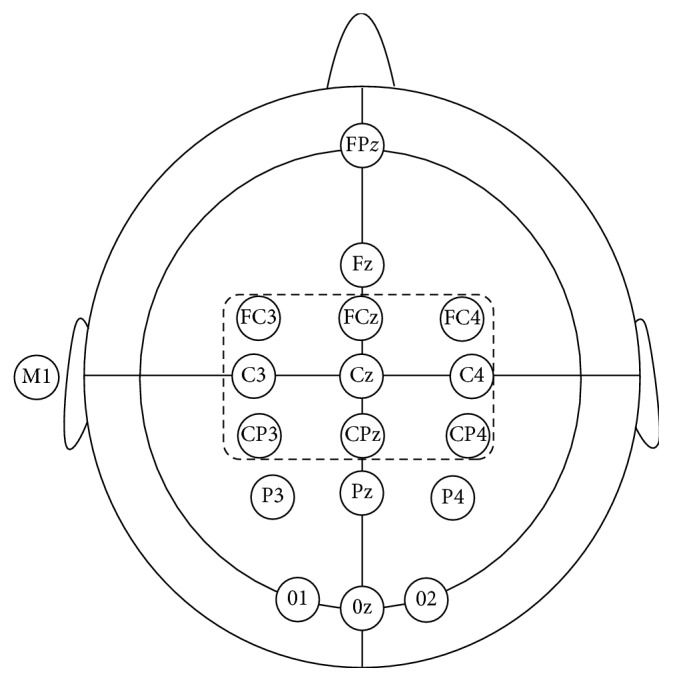
Electrode locations of EEG acquisition in 16-channel cap.

The data acquisition device for this study was a 16-channel EEG amplifier (Mipower-UC EEG Collection V2, Neural Engineering Laboratory, Tsinghua University; signal band: 0∼250 Hz; sampling frequency: 1000 Hz; A/D converter: 24 bit; without a 50 Hz frequency notch; 16-channel EEG cap (Ag-AgCl powder electrode, Wuhan Green Technologies Co. Ltd.) customized according to the ten-twenty electrode system of the International Federation of Clinical Neurophysiology)) [24, 25]. The motor cortex was covered with the 9 electrodes FC3, FCz, FC4, C3, Cz, C4, CP3, Pz, and CP4, as shown in Figure 1. The M1 on left mastoid was used as the reference electrode, and Fpz was used as the ground electrode. Additionally, it was necessary to ensure the impedance between the electrode, and scalp was less than 5 kΩ. Meanwhile, the horizontal electrooculogram (EOG) induced by eye movement and vertical EOG induced by eye blinking were recorded (the same band pass and sampling rate as for EEG, and the electrodes were positioned at the outer corner, upper, and lower sides of the eye, resp.) to exclude the trials contaminated by EOG. Although EOG collected in the study was used for other studies, independent component analysis (ICA) was used to remove the EOG artifacts mixed in EEG data in this paper.

**Figure 2:**
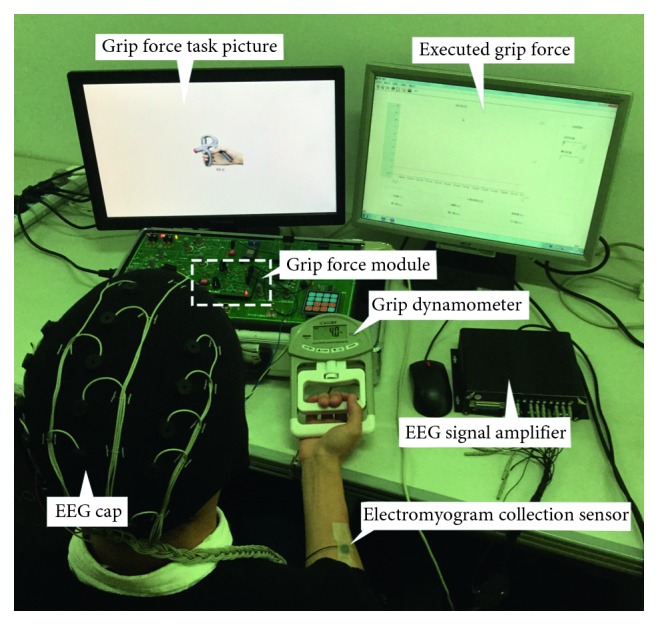
Experimental platform.

The experimental platform is shown in Figure 2. We used two computers: one for the presentation of grip force task cued pictures (using the E-prime software, v1.1) and the other for displaying executed grip force measured by the grip dynamometer (using the Pclab-800 biomedical electronic experimental box). Subjects griped according to the grip force task cued picture and the grip force value from the grip dynamometer was transmitted to the grip force module through the wire to be amplified and converted. Pclab-800 software recorded and displayed the executed value of the grip force. Meanwhile, the EEG and electromyogram (EMG) signals were obtained synchronously, and signals were amplified and saved by an amplifier.

#### 2.2.2. Experimental Paradigm and Procedure

We designed executed/imagined movement with 3 levels of grip force: low grip force (4 kg), medium grip force (10 kg), and high grip force (16 kg). EMG was collected synchronously to reflect the changes in grip force when EEG was acquired during subjects' executing/imagining movement.

**Figure 3:**
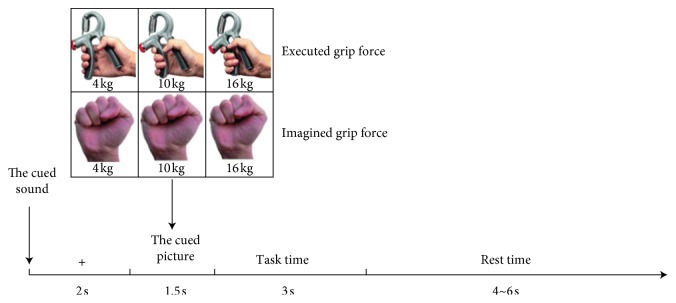
Paradigm timing in a single trial.

In the experiment, subjects were seated in a comfortable armchair and maintained a positive frame of mind. There were 3 sessions for data acquisition, each consisting of 30 trials, with each trial's timing as shown in Figure 3.

**Figure 4:**
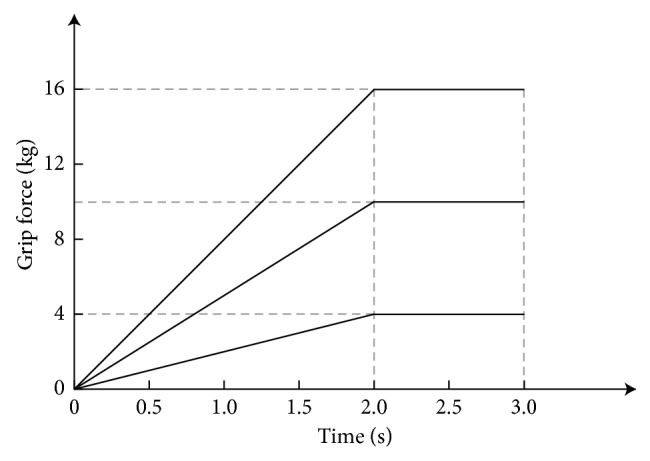
Schematic diagram of the 3 types of grip force.

A beep sound indicated the beginning of each trial, and simultaneously, a cross “+” was displayed in the center of the screen. At this point, the subjects remained quiet and relaxed; this state lasted for 2 s. Then, a grip force task figure appeared in place of the “+,” suggesting to the subjects what kind of executed/imagined grip force task should be performed next. The subjects readied for the grip force task, and this state lasted for 1.5 s. After the prompt disappeared, subjects began to perform the executed/imagined grip force movement; this state lasted for 3 s. During the task, the subjects only performed the executed/imagined grip force movement and avoided activities of other body parts, such as facial muscle activity, eye blinks, and eye movements. The changing patterns of 3 kinds of executed/imagined grip movement are shown in Figure 4. Here, 0∼2 s is the rising period of the grip force, the executed/imagined grip force linearly increased to the target grip force, and the subjects maintained the target executed/imagined grip force throughout the 2∼3 s time period. Then, subjects entered the rest state, where they could take a break, without doing any other limb movement; this state lasted for 4∼6 s.

Each trial lasted for 10.5∼12.5 s, and the total time of each session was 5.25∼6.25 min. The experiment consisted of 3 sessions with a 10 min break between them. The entire experiment finished within 1 hour, including the preparation time.

### 2.3. EEG Signal Preprocessing

EEG data preprocessing used MATLAB software (v7.11.0) [26] and EEGLAB platform (v10.0.1b). Data preprocessing included the following steps:

1. Data importing: importing raw EEG data and electrode location file.
2. Data down-sampling at 125 Hz.
3. Filtering the EEG data: an finite impulse response (FIR) digital filter filters the down-sampled EEG data at 0.05 Hz∼45 Hz.
4. Extracting segmentation of EEG data: the time period of the extracted EEG data was 0.5∼3 s (the executed/imagined grip force task began at the 0 s point when the cued picture disappeared).
5. Baseline correction to eliminate the deviation of EEG from baseline: we chose −1.5∼0.5 s as the baseline correction duration.
6. Artifact removal: an independent component analysis (ICA) plug-in for EEGLAB identifies the artifacts firstly, and then ADJUST [27] manually removes artifacts (mainly eye artifacts).
7. Re-referenced to the common average reference.
8. Superposition and average: the same condition trials corresponding to the different types of grip force (small, medium, and large) were superimposed and then averaged. Group average was calculated across different conditions or different subjects.

After the above 8 steps of preprocessing, we obtained 6 groups of EEG data (each of the group corresponding to 3 levels of executed/imagined grip force task, resp.).

### 2.4. Microstate Analysis

The multichannel EEG signals can be regarded as a series of instantaneous topographies of potentials (i.e., microstates), and it has 2 significant features [1, 6, 28, 29]: (1) most of the EEG signal can be expressed by a few topography maps; and (2) before a topography map is rapidly switched to another, it is in a dominant position and remains in the steady state for about 80∼120 ms. Compared to traditional EEG spectrum analysis, microstate analysis has 3 advantages: (1) although there were a large number of possible maps in multichannel recording, a majority of the signals (usually >70% of total topographic variance) were represented by just a few topographies [2]; (2) the topography map at any time is independent of the time before and after, and therefore, the resolution of the microstate is at the milliseconds level as opposed to the seconds level; (3) the microstate analysis can be well used both in time and frequency domain [29].Therefore, this analysis method is more suitable for the investigation of fast and dynamic brain activity than the traditional spectrum analysis.

In this study, microstate analysis was followed by the proposed processing in the early microstate study of the resting state EEG [30]. The steps are as follows: firstly, we calculated the global field power (GFP) and obtained its local maximum. Then, the local maximum was clustered into several microstates, and finally, we calculated the parameters of each microstate. EEG microstate analysis was carried out by Matlab software.

#### 2.4.1. GFP Calculation

Researchers identified points with the greatest signal-to-noise ratio (SNR) by calculating the GFP of each topography in the time series, and its calculation formula is as follows [29]:

(1) $$\begin{array}{c} \text{GFP}=\frac{\sqrt{\left(\sum_{i}^{k}{\left(V_{i}\left(t\right)-V_{\text{mean}}\left(t\right)\right)}^{2}\right)}}{k}, \end{array}$$

where *V*_*i*_(*t*) represents the instantaneous potentials of the *i* electrode at time *t* and *V*_mean_(*t*) is the mean instantaneous potentials of all electrodes at time *t*, and *k* is the number of electrodes. GFP reflects the global field power intensity of the brain at each instance, which is usually used to measure the brain response to an event or to characterize the rapid changes of brain activity.

After calculating the GFP, we obtained the local maximum of the GFP curve all points with GFP higher than the preceding and following 4 points in the time axis for the following reasons: (1) the topology between 2 local maximum on the GFP curve are relatively stable, and thus the topology of local maximum represents all topology nearby at this time point; (2) only selecting local maximum points can reduce the computational complexity of subsequent cluster analysis, without the whole GFP data at each time point being clustered; and (3) the local maximum points of GFP curve represent instants of strongest field power and highest topography signal-to-noise ratio and thus some noise components may be avoided not to cause dramatic changes of topology in this point [31].

#### 2.4.2. Microstate Clustering

To get a representative microstate, topographies at all GFP peaks were simultaneously extracted and entered into a clustering algorithm that grouped these maps into a small set of classes (microstate) based on topography similarity, without regard to the order of their appearance. The general clustering methods of microstate clustering in the literature is Atomize-Agglomerate Hierarchical clustering [6–8, 10, 28, 32–35] and K-means clustering [9, 11, 14, 29, 36–39] or improved K-means clustering; the literature [29] has proved the consistency of these 2 clustering methods, and for this paper, we adopted the K-means clustering method.

Some research in the resting state EEG defined the brain activity as 4 types of microstates [6–9, 11, 34, 35, 38, 39], corresponding to the 4 resting state networks; Meanwhile, in task states, the number of microstates is usually unknown, and to find the optimal cluster number, researchers often use the cross validation method [32, 40].

If EEG topographies at the local maximum in the GFP curve of different grip force modes are known, the change and transformation of EEG microstates in the whole grip force movement process can be compared and analyzed concretely. Cross validation is to establish a microstate model suitable for most EEG topographies with appropriate complexity and to determine the optimal number of microstates in different grip modes. This model is based on the multichannel (9 channels in this paper) EEG data corresponding to the local maximum points of the GFP curve in grip force movement of all subjects. This data set is randomly divided into training set and testing set (80% of the data set is the training set and 20% is the testing set, and each set must at least contain part of multichannel EEG data of all subject in three grip force modes). The model generated in the training process is used to predict the testing set with minimum prediction error [35]. The detail clustering process references literature [40]. In order to determine the optimal clustering number, we also calculated the pseudo *t*^2^ statistic and pseudo *F* statistic [41]. We then combined the results to get the optimal clustering number:

(2) $$\begin{array}{c} \text{pseudo}\;\;F\;\;\mathrm{statistic}=\frac{\left(T-P_{k}\right)/\left(k-1\right)}{P_{k}/\left(n-k\right)}. \end{array}$$

Here, *T* is the total sum of squares of all data set; *P*_*k*_ is the sum of the sum of the squares of each data subset in all *k* class; and *n* is the length of data set.

(3) $$\begin{array}{c} \text{pseudo}\;\;t^{2}\;\;\mathrm{statistic}=\frac{B_{pq}^{2}}{\left(W_{p}+W_{q}\right)/\left(n_{p}+n_{q}-2\right)}. \end{array}$$

Here, *B*_*pq*_^2^ is the sum of square deviation within the class after class *p* and class *q* merged; *W*_*p*_  and  *W*_*q*_ are the sums of squares of class *p* and class *q* data subset; and *n*_*p*_ and *n*_*q*_ are the lengths of class *p* and class *q* data subset.

The data sets in the formula (2) and (3) are the multichannel EEG data corresponding to the local maximum points of the GFP curve in grip force movement of all subjects, and data subset is the multichannel EEG data contained in each class.

#### 2.4.3. Calculation of Microstate Parameters

The microstate has many parameters that can reflect the characteristics of neural activity [2, 42]. The parameters used in this study are as follows: duration, which reflects the stability of potential neural assembly; occurrence, which reflects the activation trend of a potential neural source; time coverage, which reflects the occurrence percentage of potential neural source; and amplitude, which reflects the intensity of a potential nerve source. Detailed definitions are listed below [2, 6]:

(1) Duration (Dur, ms): the total time of a specified microstate (MST) in the whole analysis time period divided by the number of continuous occurrence (CON).
  (4) $$\begin{array}{c} \text{Dur}=\frac{\text{MST}}{\text{CON}}. \end{array}$$

(2) Occurrence (/s): the number of times a specified microstate in the whole analysis time period.
(3) Time coverage (TC, %): the time period of a specified microstate occurring in the whole analysis time period (WT), divided by WT.
  (5) $$\begin{array}{c} \text{TC}=\frac{\text{MST}}{\text{WT}}. \end{array}$$

(4) Amplitude (Amp, *µ*V): the sum of GFP peaks (GFPP) of a specified microstate divided by the occurrence in the whole analysis time period.
  (6) $$\begin{array}{c} \text{Amp}=\frac{\text{GFPP}}{\text{CON}}. \end{array}$$

In the majority of studies on microstates in the resting state EEG, the calculation time period of the microstate parameters is 1 s or 2 s [5, 7, 8]. However, since our research is based on microstates in the task state EEG, the calculation time period was 3.5 s (0∼0.5 s was the preparation period for the grip movement, and 0.5∼3.5 s was the execution period of the grip movement). In order to compare the microstates between executed and imagined grip movement and to improve the results significance of microstate analysis, the preparation period without executed/imagined grip force movement (0∼0.5 s) was also included in the microstate analysis.

#### 2.4.4. Statistical Analysis

In this study, the main factors affecting the microstate parameters were the 2 kinds grip force mode (executed and imagined) and the 3 kinds of grip forces (low grip force, medium grip force, and high grip force). Therefore, we conducted a 2 × 3 two-factor variance analysis and corresponding post hoc multiple comparisons (ANOVA) [24, 43] for the 4 parameters of each microstate.

In the executed grip force mode, pairwise comparison analysis of the 4 microstate parameters (duration, occurrence, time coverage, and amplitude) of 3 microstates (A, B, and C) in low, medium, and high grip forces (executed 1, executed 2, and executed 3) was made, and the relationship between microstate parameters and the executed grip force, respectively, were investigated. Then, pairwise comparison analysis of the 4 microstate parameters of low, medium, and high grip forces in executed and imagined (imagined 1, imagined 2, and imagined 3) investigated the relationship between microstate parameters and the grip force. We divided the microstates in different grip force movements into 9 comparison groups (#1: imagined 1 versus imagined 2; #2: imagined 1 versus imagined 3; #3: imagined 2 versus imagined 3; #4: executed 1 versus executed 2; #5: executed 1 versus executed 3; #6: executed 2 versus executed 3; #7: imagined 1 versus executed 1; #8: imagined 2 versus executed 2; and #9: imagined 3 versus executed 3). Furthermore, since we only investigated the microstate changes in executed/imagined low, medium, and high grip forces, we did not include the interaction terms and interaction of different factors in our statistical analysis.

#### 2.4.5. Relationship between Microstate and Band Power

Previous studies have analyzed microstates in the mu rhythm (8∼12 Hz) of EEG [11, 12, 36], but most were based on a wider frequency band, such as 2∼20 Hz [36] or 1∼40 Hz [12]. The existing research results have shown that there is no significant correlation between the 4 typical microstates and the frequency band [11]. In our study, the EEG frequency range was 0.05∼45 Hz in both executed and imagined grip force tasks whether or not the microstate is related with the frequency bands. The correlogram [11] showed the correlation relationship between variables directly. However, specific calculated data are necessary to precisely describe this relationship.

Our research was based on delta (1∼4 Hz), theta (14∼20 Hz), alpha (8∼14 Hz), beta (14∼20 Hz), and gamma (20∼40 Hz) frequency bands. Firstly, we calculated the average power of all EEG acquisition channels in each frequency band, and then we calculated the cross correlation coefficient (Pearson's *R*) [12] between the microstate and frequency band according to following formula:

(7) $$\begin{array}{c} \rho_{xy}=\frac{\sum_{i=1}^{n}\left(x_{i}-\bar{x}\right)\left(y_{i}-\bar{y}\right)}{\sqrt{\sum_{i=1}^{n}{\left(x_{i}-\bar{x}\right)}^{2}\sum_{i=1}^{n}{\left(y_{i}-\bar{y}\right)}^{2}}}, \end{array}$$

where *x*_*i*_ and *y*_*i*_ represent the GFP of the microstate and the average power of each frequency band, respectively; *i* is the length of the data analyzed (variables) (*i*=1, 2, …, *n*); $\bar{x}$ and $\bar{y}$ are the means of the 2 variables; *ρ*_*xy*_ is the correlation coefficient of the 2 variables and can measure the degree of correlation and correlation properties of the 2 variables. The value of |*ρ*_*xy*_|(|*ρ*_*xy*_| ≤ 1) reflects the correlation degree of the 2 variables, and the greater the value the stronger the degree of correlation.

## 3. Results

### 3.1. GFP Calculations for Microstates

**Figure 5:**
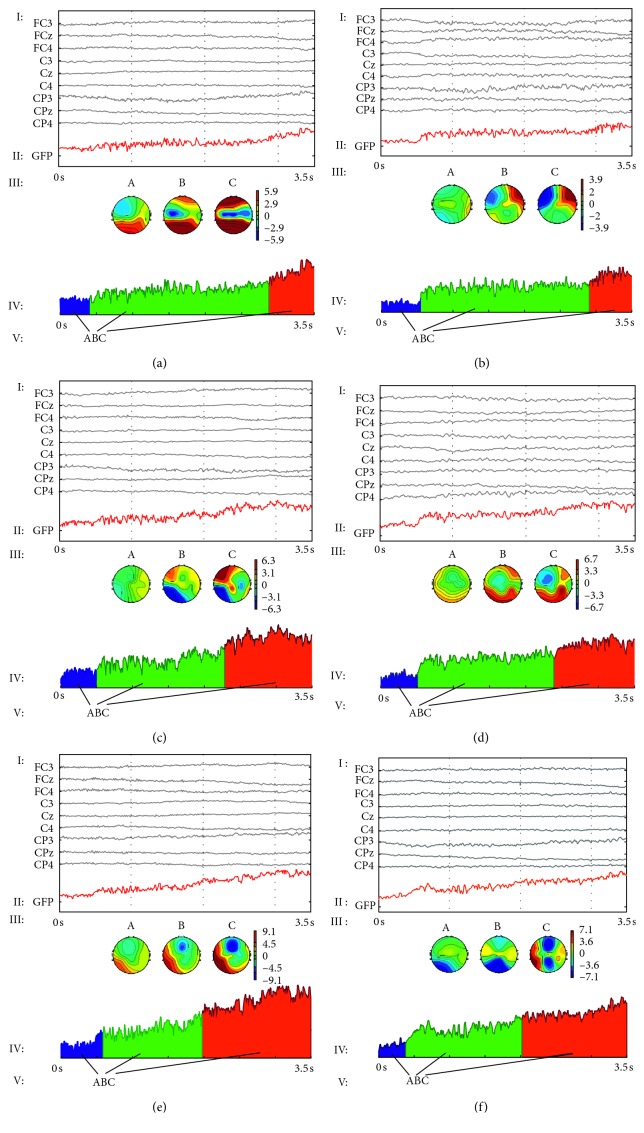
Flow diagrams of microstate analysis in executed/imagined grip forces in (a) executed low grip force movement; (b) imagined low grip force movement; (c) executed medium grip force movement; (d) imagined medium grip force movement; (e) executed high grip force movement; and (f) imagined high grip force movement.

After preprocessed using EEGLAB, EEG related to executed/imagined grip forces was analyzed by the flow diagrams of microstate analysis shown in Figure 5, where the horizontal axis represents the time (s) in executed/imagined grip movement of subjects and the vertical axis represents the voltage amplitude (*µ*V) in different grip forces and movements. Figures 5(a), 5(c), and 5(e) show the flow diagram of microstate analysis in low, medium, and high executed grip forces, respectively; meanwhile, Figures 5(b), 5(d), and 5(f) show the flow diagram of microstate analysis in low, medium, and high imagined grip forces, respectively. In these figures, I is the EEG wave of the 9 EEG channels 3.5 s after preprocessing (frequency range: 0.05–45 Hz), II is the GFP curves (thick red line) computed by formula (1), III is the topography map of microstates A, B, and C, where red is positive potentials and blue is negative relative to the reference potentials (the left mastoid M1), and the solid line is the equipotential line, IV is the region filling (blue area for microstate A, green area for microstate B, and red area for microstate C) according to microstate clustering results of local maximum in GFP curves, and V is the transition sequence of microstates in grip force tasks (i.e., syntax) [5].

Table 1 shows the average GFP value of 6 kinds of grip force tasks (executed/imagined grip force mode and low, medium, and high grip forces). It can be seen from the table that under the same executed/imagined grip force state, the average GFP value of high grip force was higher than that of low grip force, and that of medium grip force was between that of high and low grip force. Additionally, the average GFP value of executed grip force was greater than that of imagined grip force.

### 3.2. Microstate Clustering

**Figure 6:**
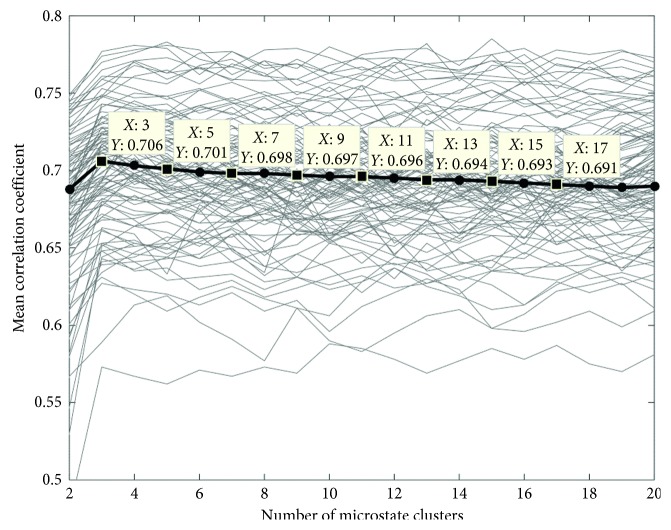
Cross validation results of the microstate model with different cluster numbers.

During the clustering process, in order to find the optimal microstate clustering number, the mean correlation of the data set with each microstate model in different microstate clustering numbers is computed. The cross validation results of the microstate model in different cluster numbers are shown in Figure 6, where the vertical axis represents the average correlation coefficient and the horizontal axis represents the number of microstate clusters. The fine grey lines denote the correlation coefficient between different training sets and the number of clusters; meanwhile, the thick black line is the average correlation coefficient of 100 times cross validations between different training set and the number of clusters. The maximum average correlation coefficient (0.706) was obtained when the cluster number was 3.

**Figure 7:**
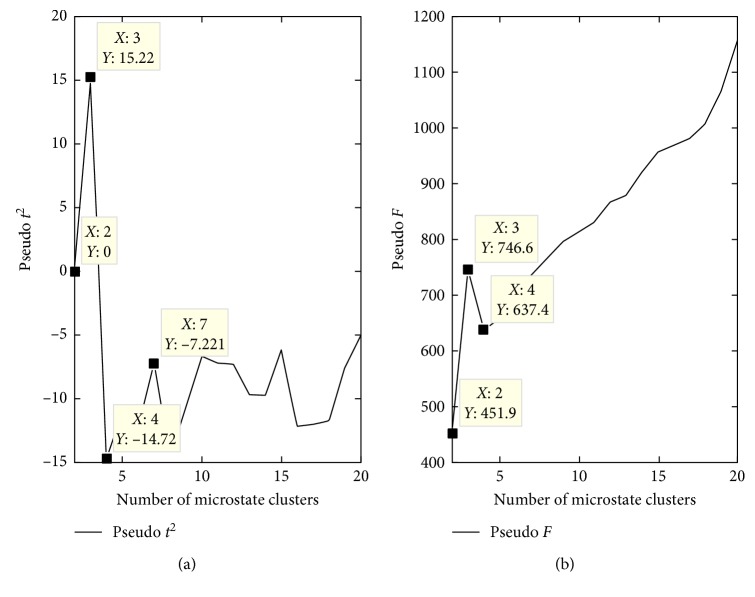
Variation of each statistic value in different cluster numbers: (a) variation of the pseudo *t*^2^ statistic; (b) variation of the pseudo *F* statistic.

To find the optimal clustering number, we also calculated pseudo *t*^2^ and pseudo *F* statistics, and the results are shown in Figures 7(a) and 7(b). When the clustering number was up to 3, both the pseudo *F* and pseudo *t*^2^ statistics reached the maximum value (pseudo *t*^2^ = 15.22; pseudo *F* = 746.6). Previous studies have shown that when the pseudo *F* statistic reaches the local peak or maximum, it means that the number of clusters is the optimal number of clusters [44]; meanwhile, the value of the pseudo *t*^2^ statistics is 15.22 > 0 when the number of clustering is 3, and the value of the pseudo *t*^2^ statistics is −14.71 < 0 when the number of clustering is 4; according to formula (3), we can see that in the process of microstate splitting from 3 into 4 class, the sum of the sum of squares within the sum of the deviations *B*_*pq*_^2^ is negative, which indicates that the optimal number of clusters is 3 from the pseudo *t*^2^ statistic perspective [45].

### 3.3. Microstate Parameters and Statistical Analysis

#### 3.3.1. Microstate Parameters

The 4 parameters of the 3 kinds of microstates related to executed and imagined grip force are shown in Table 2, where *M* is the mean value and SD is the standard deviation.

1. Duration (ms): microstate A has a short duration (496.72, 516.39, and 488.52 ms corresponding to low, medium, and high level of executed grip forces,; 519.51, 526.23, and 501.64 ms corresponding to low, medium, and high level of imagined grip forces). The duration of microstates B and C were longer compared to microstate A in both the executed grip forces (microstate B: 2008.03, 1542.62, and 1254.10 ms; microstate C: 995.25, 1440.99, and 1757.38 ms) and in imagined grip force (microstate B: 2219.67, 1762.29, and 1562.30 ms; microstate C: 760.82, 1211.48, and 1496.72 ms).

In executed/imagined grip force, the duration of microstate B decreased gradually as the grip force increased; meanwhile, the duration of microstate C increased gradually.

In the same target grip force, the duration of microstate B was shorter in the executed grip force task than that in the imagined grip force task (Force_4 kg: 2008.03 versus 2219.67 ms; Force_10 kg: 1542.62 versus 1762.29 ms; Force_16 kg: 1254.10 versus 1562.30 ms). Additionally, the duration of microstate C in the executed grip force task was significantly longer than that in the imagined grip force task (Force_4 kg: 995.25 versus 760.82 ms; Force_10 kg: 1440.99 versus 1211.48 ms; Force_16 kg: 1757.38 versus 1496.72 ms).

(2) Occurrence (/s): microstate A occurred the least of all microstates (executed: 20, 21, 23; imagined: 21, 18, 20); meanwhile, microstates B and C occurred more in the executed grip force task (B: 47, 48, 47; C: 55, 59, 65) than in the imagined grip force task (B: 35, 36, 33; C: 33, 42, 52).

In executed/imagined grip force tasks, as the grip force increased, the occurrence of microstates A and B did not significantly increase (A—executed: 20, 21, 23; imagined: 21, 18, 20; B—executed: 47, 48, 47; imagined: 35, 36, 33); however, the occurrence of microstate C did increase (executed: 55, 59, 65; imagined: 33, 42, 52).

In the executed grip force task, the occurrence of microstate B was significantly greater than in the imagined grip force task (Force_4 kg: 47 versus 35; Force_ 10 kg: 48 versus 36; and Force_16 kg: 47 versus 33, resp.), and microstate C showed the same trend (Force_4 kg: 55 versus 33; Force_10 kg: 59 versus 42; and Force_16 kg: 65 versus 52, resp.).

(3) Time coverage (%): microstate A had the shortest time coverage (executed: 14.19, 14.75, 13.96; imagined: 14.84, 15.06, 14.33); meanwhile, microstates B and C had longer time coverages in the executed grip force task (B: 57.37, 44.07, 35.83; C: 28.44, 41.18, 50.21) than in the imagined grip force task (B: 63.42, 50.35, 44.64; C: 21.74, 34.59, 41.03).

Furthermore, in executed/imagined grip force tasks, the time coverage of microstate B decreased with increasing grip force (executed: 57.37, 44.07, 35.83; imagined: 63.42, 50.35, 44.64).

In executed/imagined grip force tasks, the time coverage of microstate C increased with increasing grip force (executed: 28.44, 41.18, 50.21; imagined: 21.74, 34.59, 41.03).

(4) Amplitude (*µ*V): in executed/imagined grip force tasks, microstate A had the lowest amplitude (executed: 1.43, 1.57, 1.88; imagined: 0.75, 1.03, 1.25); meanwhile, microstates B and C had greater amplitudes in the executed grip force task (B: 2.15, 2.38, 2.95; C: 3.33, 3.68, 4.18) than in the imagined grip force task (B: 1.72, 2.16, 2.54; C: 2.33, 2.96, 3.47).

Additionally, in the executed grip force mode, the amplitude of microstates A, B, and C increased as the grip force increased (A: 1.43, 1.47, 1.88; B: 2.15, 2.38, 2.95; C: 3.33, 3.68, 4.18); in imagined grip force mode, the amplitude of microstates A, B, and C also increased as the grip force increased (A: 0.75, 1.03, 1.25; B: 1.72, 2.16, 2.54; C: 2.33, 2.96, 3.47).

#### 3.3.2. Results of Statistical Analysis

We conducted statistical analysis on 4 microstate parameters of 3 kinds of microstates with different grip forces firstly, and then conducted a two-factor ANOVA. The results are shown in Table 3, where *F* is the *F* value of ANOVA, *P* is the significant difference between different groups of data, and bolded *P* values represent *P* < 0.05, which was set as the significance level.

1. Duration (ms): the duration of microstate A showed no significant differences, while the duration of microstate B and C showed significant difference in all 9 comparison groups.
2. Occurrences: in the first 6 comparison groups (#1, #2, #3, #4, #5, and #6), the occurrences of microstate A and B did not show significant differences, but microstate C showed significant differences; in the latter 3 comparison groups (#7, #8, and #9), microstate A showed no significant difference, but both microstates B and C showed significant differences.
3. Time coverage (%): there was no significant difference (*P* > 0.05) in time coverage of microstates A and C, but time coverage of microstate B showed significant difference in all 9 comparison groups.
4. Amplitude (*μ*V): significant difference was observed in all 9 comparison groups.

### 3.4. Relationship between Microstate and Band Power

**Figure 8:**
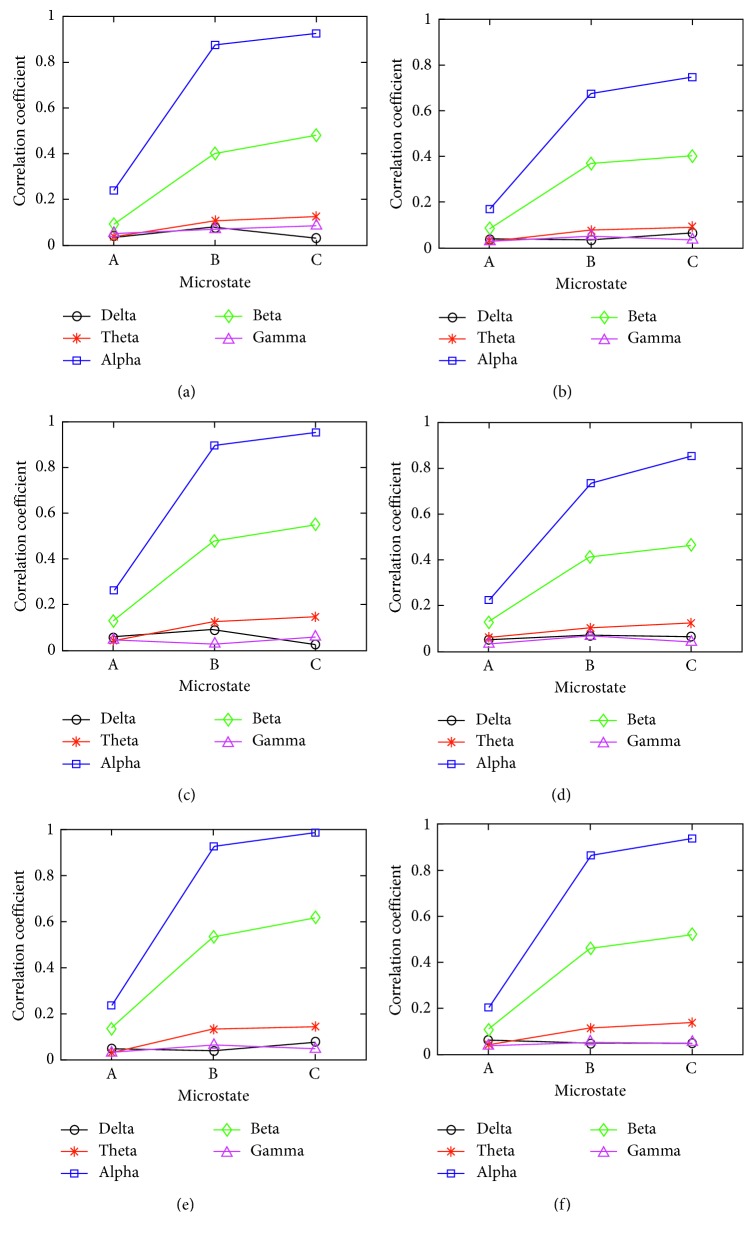
Correlograms between microstates with different grip forces and frequency bands in (a) executed low level grip force movement; (b) imagined low level grip force movement; (c) executed medium level grip force movement; (d) imagined medium level grip force movement; (e) executed high level grip force movement; (f) imagined high level grip force movement.

We first calculated the correlation coefficients of different grip force tasks between the microstate and frequency band using formula (7). Then, we averaged the correlation coefficients, and the correlogram is shown in Figure 8, where the horizontal axis represents the microstate class with different grip forces, and the vertical axis represents the correlation coefficient. Figures 8(a), 8(c), and 8(e) are correlograms between microstates in the executed grip force task and 5 frequency bands; meanwhile, Figures 8(b), 5(d), and 8(f) are correlograms between microstates in the imagined grip force task and 5 frequency bands.

## 4. Discussion

### 4.1. Microstates Related to Executed and Imagined Grip Force Tasks

As seen from Figure 5, in 3 kinds of executed/imagined grip force modes, the GFP curve of EEG increased slowly; after the multichannel EEG data corresponding to the local maximum point in the GFP curve were clustered, EEG topographies were composed of microstates A, B, and C. The microstate syntax (i.e., the conversion of microstates) [6] showed that the conversion sequence was from microstate A to B and then to C.

At the first of 0∼0.5 s, subjects were gazing at executed/imagined cued pictures on the computer screen without any executed/imagined grip force output (the preparation period); the neurons in the brain of the subjects were relatively inactive, and the EEG topology of the local maximum of the GFP curve in this time period corresponded to microstate A. The following 0.5∼3.5 s was the execution period of the executed/imagined grip force task, the subjects were engaged in motor imagery tasks at this time and the neurons in the primary motor function area of the brain were relatively active, the motor imagery became developed over time, and the two stages corresponded to microstates B and C. As can be seen from the GFP curve, the duration of microstate A was the shortest, and the amplitude of the GFP curve at this time was the weakest. Conversely, the duration of microstate C was the longest, and the amplitude of the GFP curve was the highest at this time.

The topography map of the 3 kinds of microstates is shown in Figure 5 III. When subjects performed the grip movement, the microstate gradually transitioned from A to B and C; in this transformation process, the blue color in the left hemisphere of EEG microstate topography map gradually darkened (meaning the potentials of this region decreased), and the red color of the right hemisphere darkened (meaning the potentials of this region increased). In our study, all 20 subjects were right-handed, and the collected EEG data were induced by the right-hand grip force; therefore, the potential changes in the EEG microstate topography map of the 6 kinds of grip force tasks were consistent with the event-related desynchronization/synchronization (ERD/ERS) phenomenon [46] of motor imagery.

As can be seen from Table 1, the average amplitude of the GFP curve in executed low, medium, and high grip forces was greater than in imagined grip force tasks. Moreover, the average amplitude of the GFP was highest in the high grip force task and lowest in the low grip force task. This result was consistent with that of the literature [47], in which the amplitude of cortical activity evoked by imagined movement is 25% of that evoked by executed movement. However, in some previous studies, for example, in the simple feedback task, the amplitude of cortical activity evoked by imagined movement significantly increased during subjects controlling a computer cursor and even was higher than that evoked by executed movement.

### 4.2. Microstate Clustering

Since there is no uniform standard method to determine the optimal number in clustering, we used cross validation and statistics to mutual verification. The relationship between the average correlation coefficient and the cluster number in the cross validation method is shown in Figure 6. It can be seen that the average correlation coefficient of microstate model with different number of microstates clustering fluctuated within a certain range because the multichannel EEG data were randomly divided into the training set and testing set. The motor imagery ability and the concentration degree of the subjects were different, which led to the inevitable difference of EEG in the grip force movement imagery. The maximum overall average correlation coefficient (0.706) of all microstate models was obtained when the cluster number was 3.

A good clustering method should make the number of clusters as small as possible on the premise that the data subset within the cluster is as similar as possible, so the statistical method is also used to assist in determining the optimal microstate cluster number, and we calculated the pseudo *t*^2^ and pseudo *F* statistics to determine the number of clusters, and the results are shown in Figures 7(a) and 7(b). As can be seen from Figure 7(b), both the pseudo *F* and pseudo *t*^2^ statistics reached their local maximums when the cluster number was 3. Further analysis of Figure 7(a) shows that pseudo *t*^2^ was positive (15.22) when the cluster number was 3, while pseudo *t*^2^ became negative (−14.72) when the cluster number was 4. Additionally, pseudo *t*^2^ was negative when the cluster number was greater than 4, which indicated that the interclass deviation square decreased and the intraclass deviation square increased when the cluster number increased from 3 to 4. An optimal clustering result often requires that the interclass deviation square is as high as possible, and the intraclass deviation square is as small as possible. Therefore, from this point of view, the cluster number should be chosen as 3.

These cross validation and statistical results from Figures 6 and 7 showed that the optimal cluster number was 3; therefore, the microstate clustering number in our study was 3, that is, microstates A, B, and C.

### 4.3. Microstate Parameters and Statistical Analysis

EEG microstates characterize the rapid unorganized spontaneous activity of large-scale neuronal population and reflect the brain's ability to respond to stimuli flexibly and integrate various external input information; further, changes in microstate parameters reflect different cognitive styles and the changing external environment [5].

As can be seen from Table 2, in low, medium, and high grip force tasks, the duration, occurrence, time coverage, and amplitude of microstate A were smaller than those of microstates B and C. This is consistent with the designed experimental paradigm because microstate A corresponded to the grip preparation period. The subjects did not perform the corresponding motor imagery, and the neurons in the primary motor function area of their brain were relatively inactive. Therefore, the 4 microstate parameters were less in this microstate than in the others.

Comparing microstate B with C, in the executed grip force task, with the increase of target grip force (4 kg → 10 kg → 16 kg), it can be found that the duration and time coverage of microstate B gradually decreased, while those of microstate C gradually increased. Furthermore, the amplitude of both microstates increased. Similar trends were observed in the imagined grip force task.

Comparing across the executed and imagined grip force tasks, in executed grip force mode, with the increase of target grip force, it can be observed that the duration and time coverage of microstate B were shorter, while the duration and time coverage of microstate C were longer. In the executed grip force task, the amplitude of all 3 microstates was significantly higher than in the imagined grip force task, and the occurrence of microstates B and C were significantly larger.

These results are consistent with the average value of the GFP shown in Table 1; that is, the average GFP value of executed grip force was greater than that of imagined grip force, the average GFP value of high grip force was greater than that of medium grip force, and the average GFP value of medium grip force was greater than that of low grip force.

As is shown in Table 3, the duration, occurrence, and time coverage of microstate A demonstrated no significant difference (*P* < 0.05) in the comparison with different grip force (#1, #2, and #3) and different grip force tasks (#7, #8, and #/9), but the amplitude did show significant difference (*P* < 0.05). This may be because although the subjects did not perform a specific executed/imagined grip force movement, they were affected by the task-related cued picture during this preparation period. But in fact, the subjects were not allowed to do so and should highly comply with the timing sequence of the grip force task. With the increase of executed/imagined grip force, the amplitude of microstate A increased significantly. The subconscious movement (motor preparation) during the preparation stage may be the reason for no significant difference of microstate A in duration, occurrences, and time coverage.

In summary, there were significant differences among the four microstate parameters calculated from the three microstates of EEG related to different grip force movement modes. The above analysis showed that the difference among the three microstates of EEG related to different grip force movement modes could be quantified by the microstate parameters. It is expected to lay a certain foundation for the microstate used in the feature extraction and classification of EEG related to imagined grip force movement and provide a new additional EEG feature for BCI based on motor imagery.

### 4.4. Relationship between Microstate and Band Power

As can be seen from Figure 8, in the executed/imagined grip force tasks, microstate A, B, and C were correlated with mu rhythm and beta bands but uncorrelated with the other 3 frequency bands (delta: 0.036∼0.071, theta: 0.033∼0.118, and gamma: 0.029∼0.065).

In the executed grip force task, with the increase of grip force, the correlation coefficient between microstates B and C and the mu rhythm increased (B: 0.910, 0.974; C: 0.957, 0.953, 0.978, 0.989); in the imagined grip force task, with the increase of grip force, the correlation coefficient between microstates B and C and the mu rhythm also increased (B: 0.675, 0.731, 0.863; C: 0.748, 0.85, 0.938). In the executed grip force task, with the increase of grip force, the correlation coefficient between microstates B and C and beta band increased gradually (B: 0.389, 0.412, 0.523; C: 0.421, 0.534, 0.611); in the imagined grip force mode, with the increase of grip force, the correlation coefficient between microstates B and C and the beta band continuously increased (B: 0.372, 0.415, 0.459; C: 0.405, 0.467, 0.524). Comparing the executed grip force task to the imagined grip force task, the correlation between microstates B and C and the mu rhythm and beta bands was higher.

We observed a weak correlation between microstate A and the mu rhythm (executed: 0.241, 0.263, 0.237; imagined: 0.172, 0.226, 0.204) and beta bands (executed: 0.093, 0.129, 0.136; imagined: 0.086, 0.131, 0.109). In executed/imagined grip force tasks, with the increase of grip force, the correlation between microstate A and mu rhythm and beta bands did not show significant changes; however, the correlation in the executed grip force task between microstate A and mu rhythm and beta bands is slightly higher than in the imagined grip force task.

Previous studies [43, 46] have shown that during executed/imagined limb movement, the sensory motor rhythm (mainly mu rhythm (8∼12 Hz) and beta rhythm (18∼26 Hz)) of EEG will have significant ERD/ERS phenomenon. Microstates B and C, corresponding to performing the imagined grip movement, were correlated to the mu rhythm and beta bands, which is consistent with the ERD/ERS phenomenon of motor imagined EEG. With the increase of imagined grip force, the duration, occurrence, and time coverage of the microstates decreased, but the amplitude increased; meanwhile, the 4 parameters of microstate C increased. With the development of executed/imagined grip force movement, the correlation coefficient between the corresponding EEG microstates and mu rhythm and beta band increased. This also showed that mu rhythm and beta rhythm were the dominant rhythms in EEG induced by motor imagery.

As shown in Figure 5 III, the blue in the left hemisphere gradually deepens and the red in the right hemisphere gradually deepens, and the ERD/ERS phenomenon was more obvious with the increase of imagined grip force.

In summary, the three microstates of EEG in different grip force movement modes had correlation with mu rhythm and beta bands but weak correlation with other frequency bands.

### 4.5. Study Limitations

Our study analyzed the EEG data of 9 channels in executed/imagined mode using microstates, but the relationship between the channel number and microstate was not studied. The literature [29] has confirmed the consistency results of microstates in the resting state EEG of 30, 19, and 8 channels, and fewer channels have shown more reliable results. Is there a certain relationship between microstates and the number of channels in task state EEG? Furthermore, are there a minimum number of channels for this microstate analysis method?

Our study found that there were 3 kinds of microstates in executed/imagined grip force tasks, and their parameters were different from different grip force tasks and forces. Whether this difference can be used to identify the different grip force task and force has not been discussed in our study.

## 5. Conclusions

In this study, we investigated variations in EEG activity patterns related to executed and imagined grip force using microstate analysis. We found that EEG related to executed and imagined grip force had 3 microstates, A, B, and C, which transitioned from A to B and then to C. The distribution of the scalp topography of these microstates in the left and right hemisphere was consistent with ERD. The 4 parameters (duration, occurrence, time coverage, and amplitude) of microstate A were less than those of microstates B and C. With the increase of the target value of executed and imagined grip force, the duration and time coverage of microstate B gradually decreased, while these 2 parameters of microstate C gradually increased. Furthermore, the duration and time coverage of microstate B related to executed grip force were less than those related to imagined grip force, but the duration and time coverage of microstate C related to executed grip force was greater than those related to imagined grip force. Additionally, the amplitude of the 3 microstates related to executed grip force was significantly greater than that related to imagined grip force, while the occurrence times of microstate B and C related to executed grip force were significantly greater than those related to imagined grip force. Moreover, the correlation between the microstates and frequency bands showed that these microstates were related to the mu rhythm and beta bands. This is consistent with a pronounced ERD/ERS phenomenon of sensorimotor rhythm.

This study will provide a new idea for feature extraction and classification of EEG related to executed and imagined grip force. Our future works will address the following: (1) the difference in parameters of different microstates will be used as features to classify the executed and imagined grip force tasks; (2) compared with executed and imagined grip forces, do the executed/imagined grip speeds also result in different microstates?; and (3) what is the relationship between the dynamic brain network and the transition among the 3 microstates related to executed/imagined grip force?

## Figures and Tables

**Table 1:** Average values of GFP in executed/imagined low, medium, and high grip movement.

	Executed	Imagined
Force_4 kg	2.16	1.82
Force_10 kg	2.82	2.18
Force_16 kg	3.54	3.37

**Table 2:** The 4 statistical parameters of 3 kinds of microstates with different grip forces.

Microstate parameters			Duration (ms)			Occurrence			Time coverage (%)			Mean amplitude (*µ*V)		
			A	B	C	A	B	C	A	B	C	A	B	C
Executed	Force_4 kg	M	496.72	2008.03	995.25	20	47	55	14.19	57.37	28.44	1.43	2.15	3.33
		SD	3.8	6.31	5.78	0.43	1.12	0.84	1.01	3.61	2.06	0.83	1.21	1.04
	Force_10 kg	M	516.39	1542.62	1440.99	21	48	59	14.75	44.07	41.18	1.57	2.38	3.68
		SD	2.67	7.42	6.12	0.52	0.81	1.24	0.93	2.19	1.51	0.51	1.14	0.94
	Force_16 kg	M	488.52	1254.10	1757.38	23	47	65	13.96	35.83	50.21	1.88	2.95	4.18
		SD	2.2	5.76	4.1	1.01	0.63	0.95	0.9	3.11	2.23	0.67	0.96	0.42

Imagined	Force_4 kg	M	519.51	2219.67	760.82	21	35	33	14.84	63.42	21.74	0.75	1.72	2.33
		SD	5.26	12.53	9.86	0.63	1.06	0.84	0.96	6.37	8.2	0.64	1.03	0.85
	Force_10 kg	M	526.23	1762.29	1211.48	18	36	42	15.06	50.35	34.59	1.03	2.16	2.96
		SD	4.68	10.84	10.32	0.58	0.94	1.31	0.8	9.05	10.31	0.31	0.89	1.06
	Force_16 kg	M	501.64	1562.30	1496.72	20	33	52	14.33	44.64	41.03	1.25	2.54	3.47
		SD	6.4	9.35	8.4	0.31	0.73	0.91	0.74	4.46	9.16	0.68	0.61	0.31

**Table 3:** Statistical analysis results of 4 microstate parameters in executed/imagined grip force tasks.

Microstate parameters		Duration			Occurrence			Time coverage			Mean amplitude		
		A	B	C	A	B	C	A	B	C	A	B	C
#1	*F*	0.73	4.59	4.67	0.89	1.15	5.32	0.75	4.13	4.04	19.26	25.31	28.54
	*P*	0.86	**0.031**	**0.038**	0.31	0.13	**0.02**	0.51	**0.032**	**0.036**	**0.002**	**0.001**	**0.000**

#2	*F*	0.84	6.53	15.26	1.04	0.79	23.42	0.64	16.54	14.32	41.75	38.31	46.27
	*P*	0.4	**0.012**	**0.006**	0.073	0.086	**0.000**	0.98	**0.005**	**0.009**	**0.00**	**0.00**	**0.00**

#3	*F*	0.81	5.23	4.57	0.85	1.64	15.35	0.78	4.06	3.12	30.18	15.33	20.49
	*P*	0.49	**0.024**	**0.041**	0.39	0.17	**0.003**	0.52	**0.039**	**0.043**	**0.00**	**0.003**	**0.002**

#4	*F*	1.03	3.57	5.43	0.65	0.94	7.96	0.39	3.94	2.98	1.85	3.56	4.31
	*P*	0.92	**0.041**	**0.029**	0.21	0.18	**0.037**	0.63	**0.042**	**0.031**	**0.042**	**0.024**	**0.040**

#5	*F*	0.69	11.36	16.87	0.97	1.67	19.41	0.47	21.13	17.24	14.38	23.67	17.63
	*P*	0.74	**0.021**	**0.009**	0.63	0.08	**0.004**	0.72	**0.011**	**0.013**	**0.007**	**0.005**	**0.004**

#6	*F*	0.71	4.12	6.28	1.32	0.86	11.06	0.61	6.34	5.37	2.31	1.64	4.27
	*P*	0.42	**0.037**	**0.040**	0.19	0.24	**0.023**	0.57	**0.027**	**0.034**	**0.038**	**0.029**	**0.017**

#7	*F*	0.87	4.37	4.68	0.91	4.31	13.63	0.97	10.38	4.3	26.62	31.24	30.43
	*P*	0.76	**0.034**	**0.026**	0.69	**0.036**	**0.003**	0.55	**0.017**	**0.032**	**0.001**	**0.00**	**0.00**

#8	*F*	2.19	5.34	9.72	0.94	5.14	16.13	1.32	6.85	3.83	33.21	23.48	34.39
	*P*	0.63	**0.02**	**0.013**	0.73	**0.028**	**0.001**	0.61	**0.026**	**0.04**	**0.00**	**0.001**	**0.00**

#9	*F*	1.32	4.15	4.67	1.36	6.64	8.27	0.94	3.53	4.12	29.16	32.65	35.34
	*P*	0.59	**0.042**	**0.031**	0.58	**0.016**	**0.011**	0.72	**0.041**	**0.046**	**0.00**	**0.00**	**0.00**
